# The Cost of Drugs

**Published:** 1905-11-25

**Authors:** 


### Editor's Letter-Box.

[Our Correspondents are reminded that prolixity is a great bar to publication, and that brevity of style and conciseness of statement greatly facilitate early insertion.]

### THE COST OF DRUGS.

"A Physician to a large Out-patient Department" sends us the following comments on the articles on "The Cost of Drugs" published in The Hospital on September 30 and October 7, 1905:—

"I read with much interest, not unmixed with some perturbation, the articles on 'The Cost of Drugs' which appeared in your issues of September 30 and October 7. The subject therein dealt with, whether it is economically sound policy for institutions to prepare all their own pharmaceutical preparations or buy them ready made in the open market, is a most important one; and I am quite at one with your able contributor as to the necessity for further elucidation. I find myself somewhat at variance with him, however, with regard to his conclusions, which I fear are open to much criticism, and I trust that in the interest of hospitals generally the last word has not been said upon this very important question.

"My personal feeling is that until we get down to hard facts it will be impossible to satisfactorily weigh the pros and the cons of the question, for there undoubtedly exist very divergent views. When the dispenser of a hospital tells me that he can make concentrated infusion of calumba for 3s. per gallon, where the cost of it wholesale is 1s. 2d. per lb., I can draw my own conclusions; but when your contributor writes of buying drugs for a hospital of 600 beds 'by the ton,' I am hopelessly lost. I should like to know, and I am sure this curiosity is shared by many of your readers in similar plight, what drug in the whole pharmacopœia can such a hospital profitably purchase by the ton? How long would it take such an institution to utilise a ton of any of the drugs he names, cascara, or rhubarb, or rice starch? To my mind there is still room for much to be said in answer to such statements, and I share, what I am convinced must be, the disappointment of many that someone of extensive experience has not ere this time dealt with the subject.

"But there are numerous other points which lay themselves open to criticism. There is, for example, the statement that 'it does not really matter where' this hospital is situated. I have always been under the impression that it matters very materially, because the locality of the hospital must have an important bearing on the question of expense (*vide* the subsequent remark on p. 12 that cost of materials is conditioned by *where* purchasing is done). How is it possible for any hospital pharmacist of an institution of 600 beds—not to mention a probable out-patient department open daily—to devote the time and expense necessary to travel periodically to London or other markets for the purchase of crude material? This time and outlay must enter into the calculation, together with that occasioned by having his post occupied for him during such absence.

"To say, moreover, that the purchaser in buying raw materials 'sees what he is getting' does not quite harmonise with the statement that he purchases by the ton. He can at best see only samples, and is driven to rely after all on a guarantee, 'that blessed word, like Mesopotamia, which is anodyne for anxious spirits,' and in buying raw material there is also 'unlimited scope for the cultivation of the virtue of honesty.'

"But I might multiply examples of your contributor's statements which are convertible, such as (p. 171, September 30) 'Imperceptibly you are pushed into buying an inferior article,' which applies with equal force to raw material; and (p. 12, October 7) 'Substances bought guaranteed were wretchedly defective,' which surely is no sufficient reason for the conclusion that they are always so, and that every guarantor is in the same dishonest class. Is every firm's spirit of nitre 'insipid stuff that just manages to scramble past the legal standard'? It will not do to thus argue from the particular to the general. What becomes of the thousands of gallons and tons of preparations sent out year after year by those wholesale manufacturing firms? Are they not used by pharmacists who supply satisfactorily our West End aristocracy, and who dispense satisfactorily prescriptions of medical men all over the country? Is all this material of inferior quality to what is charitably dispensed at our institutions? Is what the every-day practitioner treats his private patients with not good enough for our hospitals? In the words of your contributor, what is the 'direct inference'?

"After all, the physiological or therapeutic test is the valuable one, and what can the pharmacist do, therefore, as regards his purchases so far as these real tests go? Is he after all the person to apply the test? Am I, seeing the effect upon the patient, not to be taken into account?

"Nowadays reliable firms manufacturing on an enormous scale supply guaranteed preparations, standardised both chemically and physiologically for the use of practitioners and institutions all over the world. The plant necessary for their production involves vast outlay, for which only the enormous turnover would give a return. Methods and materials change much in a decade, and the plant of to-day might five years hence be practically obsolete. Firms of such standing supplying on such a scale would be ruined by the paltry cheating which your contributor suggests, for really that is the logical conclusion of his innuendoes. Are there no such firms which can be trusted to supply genuine preparations for the use of our hospitals? Your contributor practically says no. I should like to have some more substantial foundation than anything he puts forward to justify such an assumption.

"But as I have said, the matter is too important to be lightly dismissed. I should like some competent authority to take up the subject and give us specific details in place of vague or general statements."

